# Experiences of caregivers of children with spinal muscular atrophy participating in the expanded access program for nusinersen: a longitudinal qualitative study

**DOI:** 10.1186/s13023-020-01477-7

**Published:** 2020-07-29

**Authors:** Petra Kiefer, Janbernd Kirschner, Astrid Pechmann, Thorsten Langer

**Affiliations:** 1 Department of Neuropediatrics and Muscle Disorders, Center for Pediatrics, Faculty of Medicine, University of Freiburg, Mathildenstr. 1, 79106 Freiburg, Germany; 2 Department of Neuropediatrics, University Children’s Hospital Bonn, Bonn, Germany

**Keywords:** Spinal muscular atrophy, Nusinersen, Expanded Access program, Compassionate care program, Qualitative research, Interview study, Patient experience

## Abstract

**Background:**

Expanded access programs (EAPs) allow patients with serious, life-threatening conditions access to drugs prior to their formal approval. Despite the possible benefits for patients, EAPs present several challenges, including uncertainty regarding a drug’s efficacy and safety as well as inequities regarding access to treatment. Although the number of EAPs is growing, the experience of patients participating in EAPs has not yet been studied. In Germany, an EAP for the treatment of Spinal Muscular Atrophy (SMA) with nusinersen ran from December 2016 to May 2017). SMA is a rare, progressive neuromuscular disorder characterized by muscle atrophy and proximal muscle weakness. Insights into patients’ and caregivers’ experiences could help to improve future EAPs.

**Results:**

We conducted a prospective study using semi-structured interviews with caregivers of children with Spinal Muscular Atrophy (SMA) Type 1who participated in the nusinersen EAP in Germany. Interviews were transcribed verbatim and analyzed using an inductive approach according to the principles of content analysis. Eight families participated in the study. Their children were between 2 and 28 months old. Six children received non-invasive ventilation. Participation in the EAP marked an important turning point in the caregivers’ experiences. Their perspective changed from a severely limited life expectancy and a palliative approach to a more optimistic view including hopes for a longer life and positive development of their children. However, participating in the EAP was also challenging for caregivers in several ways. Lack of information regarding the launch of the program and the enrollment procedures caused significant uncertainty and stress among caregivers prior to the actual treatment. Further, concerns persisted that nusinersen could not be approved or that the child could be excluded due to an insufficient treatment response. Good communication and trusting relationships with medical and non-medical staff at the hospital helped caregivers cope with the uncertainties associated with the treatment.

**Conclusion:**

From the caregivers’ perspective, there was no alternative to participating in the EAP for nusinersen. All participants were positive regarding their decision to participate. However, this study suggests that developing procedures to increase speed and transparency and to ensure fairness could help to further improve the system of EAPs as a way to provide urgently needed care to highly vulnerable patients.

## Introduction

Expanded access programs (EAPs) allow patients with serious, life-threatening conditions access to drugs prior to their formal approval. Patients may enter an EAP when there is no opportunity to enroll in a clinical trial and when there is no satisfactory alternative treatment available [1, 2]. In Europe, the European Medicines Agency (EMA) provides recommendations on how to administer, distribute and use medicines for EAPs. However, the member states decide independently if, when and how they open or register EAPs [3]. In the United States, the Federal Food and Drug Administration (FDA) decides whether EAPs are launched. Between 1989 and 2016, 398 EAPs have been registered on the platform clinicaltrials.gov, offering treatment for 460 different conditions internationally. Although regulatory approval is envisaged for most drugs provided via EAPs, only 76% of the administered drugs eventually received approval by the FDA [4].

Recently, the EAP model has received increased attention and has stimulated controversial debates [5]. Supporters argue that the length of time necessary to develop a new drug (7–8 years on average [6]) is too long in the context of severely debilitating and life-limiting diseases. EAPs therefore enable patients to receive a newly developed drug even though uncertainty regarding its efficacy and safety persists. Despite the potential benefits for participating patients, concerns have been raised about EAPS and they present several challenges [7]. First, decisions concerning EAPs are made at the country-level, which means that access can differ markedly between nations. Indeed, the patient organization for Rare Diseases Europe (EURORDIS) found a time difference of more than 3 years between member states opening EAPs for a specific drug within the European Union. EURORDIS therefore demands improvements to ensure equal access to EAPs independent of a patient’s place of living [8]. Second, manufacturers decide independently to participate in an EAP and make the medicine available. Manufacturers have to weigh up costs for drug production and administration, marketing considerations, legal and regulatory aspects in their decision process [7]. Third, clinicians need to shoulder an additional administrative burden by mobilizing resources in their facility and/or supporting patients and caregivers in the enrollment process [9].

In 2016, EAPs for nusinersen to treat patients with Spinal Muscular Atrophy (SMA) Type 1 were initiated in several European countries and the US [10]. SMA is a progressive neuromuscular disorder characterized by muscular atrophy and proximal muscle weakness. SMA has an incidence of 1:10.000 and is the most common genetic cause of death in children. Without treatment or respiratory support, patients with the most severe phenotype (SMA Type 1) usually die within the first 2 years of life [11]. Prior to the availability of drug treatment for SMA, there was no hope for parents that the clinical condition of their child would improve because the natural course of the disease is invariably progressive. They were faced with complex decisions concerning respiratory support or feeding via percutaneous endoscopic gastrostomy [12–14]. Nusinersen is a drug which might change the course of the disease significantly. The placebo-controlled phase III trial was terminated early after interim analysis showed that patients in the treatment group showed positive motor development and better survival [15].

Patient organizations have been a driving force in the development of EAPs since the AIDS epidemic in the 1980s. However, from a patients’ perspective, EAPs are associated with uncertainties on multiple levels, including the safety and efficacy of the drug, the access to an EAP (where and when) and treatment continuity after the end of the program. Despite the growing number of EAPs, the experience of patients participating in EAPs has not yet been studied. The aim of this study was to explore the experiences of caregivers whose child was diagnosed with SMA type 1 and who participated in the EAP for nusinersen in Germany. We anticipated that caregivers whose children had a life-threatening disease and who (suddenly) gain access to a possibly life-saving treatment live through a challenging experience, which might change over the course of the program. We therefore conducted a prospective qualitative interview-study in which we addressed the following research questions:

1. How did caregivers experience the process of getting access to and receiving treatment within the EAP for nusinersen?
2. How did caregivers cope with the limited information regarding benefits and risks of the treatment?

Insights into the experiences of patients and caregivers participating in EAPs could help to further improve the policies and processes associated with EAPs.

## Methods

For this study, the methods are presented according to the consolidated criteria for reporting qualitative research (COREQ) checklist [16]. We conducted a prospective, qualitative study using repeat interviews at a university medical center in Southwest Germany. It is one of seven centers where nusinersen was offered within the EAP in Germany. The hospital is a large reference center for muscular disorders in Germany and member of the European Reference Network for Rare Diseases.

### Background information regarding the EAP of nusinersen in Germany

Prior to the EAP, the effect of nusinersen had been investigated in a phase III clinical trial for patients with SMA Type I [15]. This trial was terminated early in August 2016 after interim analysis showed higher survival and better motor development in the treatment group. Two months later, the nusinersen EAP was approved by the Federal Institute for Drugs and Medical Devices on October 14, 2016. In November 2016, the first patients received treatment with nusinersen at our hospital. Nusinersen received approval through the EMA on June 1, 2017 and the EAP was closed on May 31, 2016.

Shortly after the approval of the EAP, participating centers and the patient organization “German Society for Muscle Patients” (DGM) set up a strategy to inform patients and direct inquiries. Caregivers were advised to obtain information about participating centers and details regarding the treatment through the DGM. To this end, the DGM established an information bureau, which sought to help direct interested families to the medical centers and avoid duplicate inquiries. Initially three centers with special expertise in neuromuscular diseases offered treatment with nusinersen; the number of centers later rose to seven.The centers collectively published an online statement indicating that decisions about whether and when a patient could be treated could only be made on a case by case basis [17]. The statement also mentioned that young and less affected patients with SMA Type 1 would be given priority because the evidence for nusinersen’s effectiveness was strongest for this group. An additional prerequisite for enrollment was that patients had to apply to their health insurance to cover the costs for drug administration. The statement did not include information regarding the regulatory approval procedure via the EMA or options for ongoing treatment after the end of the EAP.

### Data collection and analysis

Data were collected December 2016 through May 2017. Interviews were conducted with the patients’ caregivers while patients were hospitalized for the treatment with nusinersen. The treatment with nusinersen followed a schedule with predefined treatment days (days 1, 14, 28, 63, 180 followed by injections every 4 months). Patients were admitted to the hospital for each treatment for an average stay of two nights, sincenusinersen was administered intrathecally by a lumbar puncture requiring analgosedation and/or continuous monitoring.

The average age of patients was 15 months (range: 2–26 months). Most patients required non-invasive ventilation and tube-feeding due to their advanced stage of the disease. The patients’ characteristics are shown in Table 1.

**Table 1:** patients’ characteristics

Total number of patients	8
Female	4
Average age at enrollment	15 months (SD 7.7)
Non-invasive ventilation	6
Feeding tube	5

As the goal of the study was to reconstruct the caregivers’ experience in its development over time, we decided to collect data at three points. We conducted two semi-structured oral interviews: the first on day 14 or day 28 (t0) and the second on day 180 (t2). We also performed a written interview at day 63 (t1). The interview-guide was developed in a multi-disciplinary group, including representatives from child neurology, pediatric nursing and health care research. Interviews consisted of open-ended questions focusing on the experience of participating in the EAP. In each interview we asked caregivers about the current condition of their child and how participating in the EAP impacted related to the child’s and families’ wellbeing. These questions were repeated during the three interviews, whereas others changed over time. Particularly in the first interview, we explored how caregivers got to know about the EAP and what they were expecting from it. In contrast, in the third interview we asked questions reflecting on how caregivers felt about the EAP when they were looking back and what they thought could have been handled differently. The interview guides are provided as additional material online.

One of the authors (PK) conducted the interviews. PK is a female registered nurse (RN) with experience in child neurology, palliative care and qualitative interviewing. She did not have a therapeutic relationship with interviewees prior to or during the study period. Interviews were digitally recorded and transcribed verbatim. The database consisted of 348 pages transcripts equaling 654 min of interviews.

PK and TL (MD, male) performed the primary data analysis following an inductive approach according to the standard principles of content analysis [18, 19]. During the first phase of the analysis, transcripts were analyzed by open coding. PK assigned preliminary themes and subthemes were assigned to the passages in the transcripts that seemed relevant to the research questions. Next, PK and TL independently reviewed all codes and proposed refined themes and subthemes. Both investigators compared their themes and discussed commonalities and differences. In case of differences, they revisited the original data to refine their interpretations. For each theme, a detailed summary was written to capture nuances and possible relationships to other themes. In the second phase open codes were grouped when they referred to similar themes, e.g. “information”. In the third phase, these themes were weighted in a discursive process between PK and TL regarding their relevance for the research questions and hypotheses regarding the relationship of the themes were formulated.

At three stages of the analytical process, interim results were presented to an interprofessional group of researchers to ensure face validity of the emerging themes, to maintain a high level of intersubjectivity and to avoid personal bias.

## Results

Eighteen families participated in the EAP at our hospital. All caregivers were eligible to participate in the present study. Participation was voluntary and participants received no incentive. Eight families were enrolled in the study. All eight mothers participated in the t0 and t2 oral interviews and the t1 written interviews. In addition, three of the fathers participated in interviews at t0 and two of the fathers participated in interviews at t2. Reasons for non-participation in the study were problems conducting an interview during the hospital stay (some caregivers had to stay with their child continuously), limited German proficiency and lack of interest in participating in the study. One family was considered to be too emotionally burdened to participate in the interviews.

The caregivers’ experiences regarding participation in the EAP were embedded in their overall experiences of caring for a child with a complex chronic neurological condition. This overall experience has begun prior to the EAP when children developed their first symptoms or when SMA was first diagnosed and continued after nusinersen received regulatory approval. However, in this study we focused on those experiences that could be directly linked to the EAP. We identified emergent key themes in our interviews which are presented in the following section. The first 4 themes are organized in a successive order as they were experienced by the participants. Paragraphs 5 and 6 refer to themes without a clear time reference.

### Life with SMA prior to the EAP

Prior to the EAP caregivers were faced with an invariably progressive and ultimately fatal disease.

*“For the past 1.5 years, since [the child] was born our lives were all about her. It was all about spending time with her. I always had in mind: We only have those 2 years.” (5a 380)*

*“We have already thought about funerals. He [the child] really loves trees. When he couldn’t sleep I would take him in the baby buggy and walk him in the woods for an hour. This is why we decided to choose a “peace forest” once the hour comes. There he will hear the leaves rustling.”(2a 280)*

*“It was so horrible to see death before your eyes; having to watch my child die.” (7b 33)*

### Experiences prior to the enrollment in the EAP

After the initial news that an EAP was in preparation, parents experienced a phase characterized by a high level of uncertainty. Participants described their concern that the EAP would not be launched at all or that it could be too late for their child to benefit. Some participants gave insight in the time of waiting in agony:

*“That was a horrible time. I think it was almost worse than waiting for the diagnosis.” (5a 163)*

*“We were hoping for a fast approval. Because every day counts! [ …] that he doesn’t deteriorate and stays stable as he was.” (6a 140ff)*

While all interviewees described their experience of impatiently waiting, others tried to expedite the approval process, as one father described:

*“The study [phase III trial] was terminated. The application [for the EAP by the pharmaceutical company] was submitted. They [the pharmaceutical company] published that. Next, nothing happened for four weeks. I asked other parents in our WhatsApp group if we should send inquiries to the authority as a group and make the delay public via Facebook. But people didn’t follow. So, I decided to pretend to be a doctor and call myself. After I got the right extension number, I called the office several times. Then I found out that the delay was because the consent form had to be finalized. I asked them: “Seriously? You are fighting over a piece of paper on the back of dying children?” Two days later, the EAP got approved.”(2a 1134 ff)*

After the formal approval of the EAP caregivers experienced another phase of uncertainty because the route for participating in the program was not yet clearly established. Participants experienced the time between the approval of the EAP and the actual start of the treatment as agonizing, being torn between hope for treatment and fear of not being accepted.

*“Every day from September when I came back from work I asked “Have they called? Have they called? Have they called?” But they never called. […] And by the beginning of November they said: “You can come and the health insurance agreed, too.”(6a 150ff)*

### Experiences after enrollment in the EAP

After patients were enrolled in the EAP, the caregivers’ experiences changed on multiple levels. First, the clinical situation of most patients stabilized which had a reassuring effect on caregivers.

*“We are really happy about [the child]‘s development. He likes to sit on my lap and moves his hands and arms. Right now, we are a little concerned because of a respiratory infection. But this concern is nothing compared to the irrepressible fear we experienced before.”(3b 13ff)*

Second, participating in the EAP gave caregivers a reason to hope for their child – to hope for a positive development and more independence.

*“Head control and sitting [of the child] – this is my realistic dream.”(4a 356)*

*“We are not hoping she can walk or fly or anything else. We do not want to become a super-ballerina. But maybe she can pull herself up one day, or walk a few steps. That she can go to the toilet herself. Then we’re happy, that’s it.”(7a369ff)*

Third, these positive effects had a positive impact on the caregivers themselves.

*“After the EAP everything calmed down a bit. To be honest, before, our marriage was at times a little critical.” (5b 882ff)*

Despite the overall improved outlook, several interviewees mentioned areas of persisting uncertainty and fear. These areas included the availability of nusinersen. Some caregivers were worried that nusinersen could not be officially approved in Germany. Other voiced concerns that treatment might be withdrawn if their child did not show sufficient progress.

*“I am scared the approving authority could say: Treatment is only available for certain types, or a number of SMN 2 – copies or so. That they could limit access in an extreme way and that she would not be able to participate any more. That is my biggest fear. It would be like a second lethal diagnosis!” (4a 362)*

*“Every time we are having the physiotherapy test [standardized test for SMA (CHOP Intent) which has been used in phase III studies* [20]*] I am thinking “Mhm” - hopefully, she will not be kicked out of the program because she does not show the results they are hoping for.” (5a 970 ff)*

### Life with SMA with treatment with nusinersen

After the EAP was launched, the outlook for life for interviewed caregivers changed significantly. The children’s clinical situation stabilized and some parents noticed signs of positive motor development. The changes caregivers perceived in their child altered their own experiences as parents.

*“Last March, we went to a baby flea market to buy summer clothes for her [the child]. This felt so good and was a huge leap forward. In the past, we only bought clothes in her current size because we were scared she might not get the chance to wear bigger clothes.” (1b 46ff)*

*“Everything has changed, 100%! The feeling of dying is gone. Her breathing is great, 98% and her voice is getting louder!” (7b 15ff).*

The radical change from a very limited life expectancy to new perspectives for life was also challenging for some caregivers.

*“The news that a drug treatment would be available soon caused quite a mess in our heads at first.”(2a 97 ff)*

### Supporting and aggravating factors influencing the adjustment process

All interviewed caregivers valued the opportunity of the EAP to treat their children as it meant they had a chance to survive and develop. However, this new opportunity required them to make significant adjustments in their perspectives on the lives of their children and themselves. This change can be described as moving from a palliative to therapeutic approach. Through this period of adjustment, caregivers experienced factors they identified as supportive and others thataggravated their situation. An overview of those factors is presented in Table 2. Interestingly, the supporting factors refer to the relationships with doctors and the care team that families were able to develop during their inpatient stays. In contrast, factors aggravating the caregivers’ situation can be attributed to authorities, organizations and (unknown) procedures. Seemingly, the perceived lack of transparency and/or contact persons made it difficult for caregivers to act.

**Table 2:** Supporting and aggravating factors influencing caregiver’s adjustment process

Supporting	Good information	*“We got all the information [at the Medical Center]. We felt well informed. Really. We spoke about that he will not become a soccer playing child, not going to happen. That’s okay. And that you can’t really say what will happen. The possible side effects were presented very, very clearly in the information sheet.” (2c 179ff)*
	Good relationships	*“They [doctors on the floor] are asking how it is going? Do you think it is working? How did he do after the puncture? It is a good mixture, they are hopeful, too. But, they are also realistic. They are listening neutrally. No one is promising too much, but they are not pessimistic either.”*
	Recurring processes during treatment in the hospital	*“The first time it was like “is it going to work? How will he tolerate the medication? The lumbar puncture? Then it worked, and the second time again. That was such a liberating feeling! And now it just works. My wife knows the hospital, I know the hospital. Everyone knows what to do.”(2aa 894ff)*
Aggravating	Lack of information regarding EAP launch	*“No one could say how long it would take to approve the program. And we didn’t know how [the child] would do when it started.” (1a 74ff)*
	Lack of information regarding enrollment and criteria	*“There has to be someone who oversees the whole program and who takes some leadership, that not everyone can do what he thinks is right. Well, I find this hurtful for parents.”(5a 941ff)*
	Concerns regarding continuation of treatment	*“If something happens, like a severe side effect for example, they can close down the whole program, just like that.” (5c 1322ff)*

### Solidarity and justice

Several participants expressed their empathy with patients who could not participate in the EAP or who might only get their treatment later.

*“All children should get a chance – all children on this earth.” (7a, 1153)*

*“I find it really difficult when I see children who are doing worse than [our child]. I mean it is clear that we want this for [our child]. But then you start thinking: Are we taking a place for a child that might even die because they do not get the treatment fast enough?” (3a 312)*

The question of which children should be treated first was also subject of discussions in the social media group, as one participant reported

*“Everyone thinks their child should come first. One argued [in the WhatsApp Group] that those who were older and who had been fighting for so long should be treated first . Whereas I say those who are still able to do something should be treated first because they have a chance that some function comes back. […] Anyway, I wrote she should be careful what she writes. We are all in the same boat. Everyone wants the best for their child and in the end it will be the doctors who decide. We must not tear ourselves apart.” (4a 567ff)*.

## Discussion

In this longitudinal qualitative study we analyzed the experiences of caregivers whose children with SMA 1 participated in the EAP nusinersen at a university medical center in Germany. The opportunity for an effective treatment marked a life-changing turning point for the interviewed caregivers. Their perspective changed from a severely limited life expectancy and a palliative approach to a more optimistic view including hopes for a longer life and a positive development of their children. Despite the uncertainties associated with the treatment, for participants in our study there was no alternative to the decision to treat the child. However, participating in the EAP was also challenging for the caregivers in several ways. Most importantly, lack of information regarding the launch of the program and the enrollment procedures caused significant uncertainty and stress among caregivers prior to the actual treatment. During treatment within the EAP concerns persisted: that nusinersen would not be approved or that the child might be excluded due to an insufficient treatment response. Caregivers mentioned that good information and good relationships with medical and non-medical staff at the hospital helped them to cope with the uncertainties associated with the treatment. Participants also raised concerns regarding equal access to the treatment for patients with different degrees of disease severity and/or country of origin.

To the best of our knowledge, this is the first study analyzing the experiences of caregivers whose children participated in an EAP. In the following section we will discuss 3 aspects which seem of particular significance: emotional burden, decision-making and challenges in providing care.

### Emotional burden

Some of our findings regarding the caregivers’ emotional burden are supported by other studies. Yang et al. analyzed the experience of an anticipatory loss of parents who care for a child with SMA Type 1 [21]. Their results are comparable to the experiences our participants reported when they referred to the time prior to the enrollment in the EAP. In that study, parents emphasized the meaning of spending time with their child and to “live in the moment” as well as the need to enrich the child’s shortened lifetime and an overwhelming helplessness. Other studies have described the considerable psychosocial burden for parents of caring for children with SMA as a life-limiting illness [22–25].

### Decision-making

The way that patients and caregivers of patients with SMA come to treatment decisions with regard to new treatment options has been subject to two recent studies. Both were conducted among patients with SMA Type II and III and parents of patients with SMA Type 1. Thus they represent a more heterogeneous sample than our study. Paccione et al. interviewed thirteen patients after the approval of nusinersen by the US Food and Drug Administration (FDA) with regard to their perspectives and expectations. Five were not interested in receiving treatment. The others either received treatment or were still in the process of decision-making. The study showed that although no other treatment had been available at the time of the study, participants weighed the potential benefits and risks of the treatment carefully. The authors concluded that better data on treatment effects of nusinersen in different types and states of the disease are needed to support an informed decision by patients [26]. Another survey by Cruz et al. investigated the cost-benefit evaluations of patients with SMA and parents of patients with SMA [27]. Using hypothetical combinations of potential benefits and risks, the authors confirmed that patients weigh their options carefully and need good information to come to a decision. Both studies emphasize the role of clear and good information for patients regarding a new treatment – even if there is no other treatment option available.

### Challenges in providing care

Participants in our study expressed that lack of transparency pertaining to the access to treatment, selection criteria and continuation of treatment after the end of the EAP caused considerable stress. These findings resonate with the work of several authors who have analyzed the challenges associated with EAPs from an organizational and ethical perspective. Whilst a comprehensive ethical discussion is beyond the scope of this paper, we would like to highlight three important aspects which resonate with our findings: 1) informed consent, 2) fair patient selection and 3) institutional support for patients and physicians.

1. Informed consent: Borysowski et al. point out that informed consent in EAPs can be challenging to obtain for two reasons [28]. First, data on a drug’s efficacy and safety can be limited due to its (early) developmental stage. Second, patients participating in an EAP often have a life-threatening condition and the drug provided through the EAP might be their only treatment option. Patients may thus overestimate the benefits and underestimate the risks of the treatment. Therefore effective counseling for patients and caregivers by physicians in the process of informed decision-making seems of particular importance. In our study, several caregivers emphasized the value of good information regarding the treatment that they received from their pediatric neurologists once they were enrolled in the program.
2. Fair patient selection: In EAPs, manufacturers can decide what quantities of a drug they provide which raises the question who should receive the treatment [29]. Decisional conflicts can also arise on medical grounds when inclusion and exclusion criteria for the treatment or queuing procedures for prioritizing patients have to be defined [30]. In this study, caregivers reported a high level of stress when they were waiting for official approval of the EAP and for their acceptance in the program at the Medical Center. From their perspective, the approval process and the criteria for enrollment were not transparent which they experienced as disempowering.
3. Institutional support for patients and physicians: Caplan et al. report on the experiences of a Compassionate Use Advisory Committee (CompAC) to address some challenges associated with an EAP for the treatment of multiple myeloma with daratumumab [31]. An international group of experts from medicine, ethics and patient advocacy provided support to a manufacturer with reviewing patient requests and make recommendations regarding the treatment with the drug. The overall experience was positive, as the committee was able to make recommendations independently and quickly. Participants in our study addressed the patient organization DGM for information regarding the nusinersen EAP. However, decisions regarding EAP enrollment were made in each medical center independently. Thus caregivers had to deal with different institutions and uncertainty persisted. Participants also voiced concerns regarding the perceived injustice of the fact that patients with SMA from other countries did not have a chance to receive treatment. Although the daratumumab EAP shows important differences with the nusinersen EAP, e.g. diagnosis and age of patients, it seems that an independent advisory board could help to improve the situation for patients and caregivers by facilitating structured, interdisciplinary discussions with transparent decisions across several institutions or even countries. However, such a committee requires funding to provide the necessary resources.

This study has several limitations. First, participants were recruited in a single center. Interviews with caregivers of patients who received treatment at another center might have helped to explore a wider range of experiences related to the EAP. Second, it seems likely that caregivers’ experiences within the EAP were positively influenced by the positive effect of the treatment on their children’s health status. If treatment with nusinersen had shown no effect this could have reflected on the caregivers’ experiences within the EAP. Therefore it seems worthwhile to study experiences of caregivers whose children participate in EAPs with less effective treatment in order to differentiate the two influences in the future. Third, we interviewed caregivers who were not patients themselves, and caregivers’ experiences should not be conflated with those of patients. For these reasons, one cannot generalize our results to patients participating in other EAPs. However, we argue that it can be assumed that patients who participate in EAPs generally have severe, life-threatening conditions and a shortened life expectancy. They therefore represent a particularly vulnerable group of patients. Further, they have to make decisions on a less-than-optimal availability of information regarding the risks and benefits of the treatment. For these two reasons, policy makers, manufacturers and clinicians should strive to develop procedures that guarantee

- that decisions regarding the EAP approval are made in a transparent way and as swiftly as possible to minimize uncertainty and
- that enrollment criteria and procedures are developed and communicated in a coordinated and unambiguous way to avoid duplicate requests and to promote fairness among patients.

Engaging patient organizations in the development of such procedures could help to take these considerations into account on a policy level.

## Conclusion

Getting access to nusinersen treatment through the EAP was invaluable to the caregivers interviewed in this study. However, lack of information and uncertainty regarding the approval of the EAP and the enrollment process created considerable uncertainty and stress for caregivers who were already in a vulnerable position. Developing procedures to increase speed and transparency and to ensure fairness could help to further improve the system of EAPs as a way to provide urgently needed care to vulnerable patients.

## Data Availability

The datasets analyzed during the current study available from the corresponding author on reasonable request.
